# Application of Eye-Tracking Technology in Assessing Binocular Vision Function in Paediatric Populations: A Scoping Review

**DOI:** 10.3390/jemr19020040

**Published:** 2026-04-17

**Authors:** Ong Huei Koon, Noor Ezailina Badarudin, Byoung-Sun Chu

**Affiliations:** 1 School of Graduates Studies, Management and Science University, Shah Alam 40100, Selangor, Malaysia; 2 Department of Optometry Rehabilitation and Well-Being, Faculty of Health and Life Sciences, Management and Science University, Shah Alam 40100, Selangor, Malaysia; 3 Department of Optometry & Vision Science, Daegu Catholic University, Gyeongsan-si 38430, Gyeongbuk, Republic of Korea

**Keywords:** anomalies, binocular, children, eye-tracking, paediatrics, vision

## Abstract

**Background:** This review discusses the application of eye-tracking technology in the detection and monitoring of binocular vision anomalies among children. **Methods:** A scoping review using PRISMA guidelines was conducted through Scopus, ScienceDirect, and PubMed using the keywords “eye-tracking,” “binocular,” “vision,” “anomalies,” “paediatrics,” and “children” from 2015 to 2025. Studies excluded were not written in English, did not apply the eye tracker as a research tool, involved an ineligible population, or involved non-human subjects. **Results:** The search strategy identified 77 citations, yet only 14 studies met the inclusion criteria. This review revealed a variety of binocular vision anomalies detectable through eye-tracking systems, along with the specific models and parameters employed in these assessments. Application of eye-tracking technology in diagnosing conditions such as strabismus and amblyopia demonstrated potential for improved accuracy and early detection. **Discussion:** Eye-tracking technology demonstrates considerable potential for the detection and monitoring of binocular vision anomalies in children, particularly as a non-invasive method for early screening, thereby strengthening its clinical applicability. By assessing fixation stability, saccadic movements, and vergence responses, eye-tracking allows for the early detection of subtle visual anomalies, especially in the paediatric population. **Conclusions:** Eye-tracking technology represents a valuable advancement in paediatric vision care, enabling the more objective and earlier detection of binocular vision anomalies in the paediatric population.

## 1. Introduction

Eye movements are fundamental in maintaining binocular vision and can be classified into two primary categories: gaze stabilisation and gaze shifting. Gaze stabilisation involves fixation, the Vestibulo-Ocular Reflex (VOR), and optokinetic nystagmus. Gaze shifting is achieved through saccades, VOR cancellation, smooth pursuit, and vergence [1]. Saccades are fast, conjugate eye movements that shift gaze from one point to another horizontally, often described as the rapid transitions between fixations. In contrast, smooth pursuit allows for the steady tracking of moving targets. Although saccades and smooth pursuits are generated monocularly at the neural level, their coordination in normal binocular vision follows Hering’s Law of Equal Innervation, whereby monocular or disconjugated eye movements are explained as the mathematical combination of version and vergence movements [2]. Precise saccadic control is essential for guiding motor responses in activities such as reading, writing, and sports. Abnormal saccadic eye movements may indicate binocular vision disorders, amblyopia, strabismus, or underlying neurological conditions. Fixation is crucial for stabilising and fixating an image on the retina for 100–300 ms [3,4,5]. It consists of microsaccades (which occur once or twice per second), periods of relative stability known as eye drifts, and tremor, which is a small and rapid oscillation [6]. When the fixation of each eye is not perfectly aligned while the brain attempts to maintain fusion, this is referred to as fixation disparity [7]. Such disparity increases vergence demand and disrupts binocular fusion, potentially leading to convergence excess or convergence insufficiency over time [7].

Binocular Vision Anomalies (BVAs) are conditions that affect one or both eyes by disrupting their ability to function as a coordinated unit. These anomalies interfere with both the motor and sensory systems, resulting in an inability to fuse two distinct retinal images into a single, clear, and three-dimensional (3D) perception. A healthy binocular system relies on a complex interplay between the motor system (which controls vergence and eye movements) and the sensory system (which processes visual input in the cortex) [7]. Both systems are crucial in ensuring precise eye alignment and coordination between both eyes, while simultaneously preventing retinal image fading [6]. However, when this coordination fails, the resulting BVAs can significantly impair a child’s neurodevelopment, affecting “eye–limb” coordination, reading fluency, and academic performance [8].

BVAs can be classified as strabismic and non-strabismic. Strabismus (also known as tropia) is a manifest misalignment of the visual axis, which can lead to diplopia (double vision) or sensory suppression. Sensory suppression is a compensatory mechanism where the brain ignores input from the misaligned eye to avoid visual confusion [9]. The anomalies can be associated with an asymmetric, variable, and subnormal convergence response [9]. For example, amblyopia is clinically defined as a defective reduction in visual acuity in one or both eyes that persists even after the correction of refractive errors or the removal of any pathological obstacles to vision [10,11]. It commonly arises from abnormal visual experience during early visual development and most frequently due to strabismus or anisometropia. Strabismic amblyopia occurs due to a deviation of one eye causing the brain to receive two images with different spatial projections, one of which comes from the deviated eye. Consequently, the brain is unable to properly combine the two images to produce stereoscopic vision, leading to reduced visual development in the affected eye. In contrast, anisometropic amblyopia arises from a significant difference in refractive error between the two eyes, causing the brain to preferentially rely on the clearer eye and resulting suppression of the blurrier eye [10]. This imbalance can lead to both functional and anatomical changes in the primary visual cortex (V1). Non-Strabismic Binocular Vision Anomalies (NSBVAs), referred to as heterophoria (or phoria), represent the latent deviations of the visual axes. Under normal viewing conditions, the visual axes of both eyes remain aligned on a fixation point. Still, deviation becomes evident when the eyes are dissociated (e.g., when either eye is covered) [12]. Although less visually apparent than strabismus, NSBVAs can induce significant “visual stress” as the motor system must exert continuous effort to maintain binocular alignment.

Clinically, binocular vision function is evaluated through several key measurements. Vergence refers to the disjunctive movements of the eyes that maintain single binocular vision, and abnormalities may manifest as strabismus (a manifest deviation) or phoria (a latent deviation controlled by fusion) [7]. Vergence performance is commonly assessed through vergence amplitude (the maximum ability to converge or diverge), vergence accuracy (often evaluated using fixation disparity measures), and vergence facility, which assesses the speed and flexibility of vergence responses under prism demands, typically using 3 Prism Dioptres (PD) Base-In (BI) and 12 PD Base-Out (BO) flippers [7]. These measurements provide insight into the efficiency and stability of binocular alignment during sustained visual tasks.

Since abnormal eye movements are often the primary indicators of these conditions, eye-tracking technology offers an objective and quantifiable approach to support clinical evaluation. Eye tracking refers to the process of electronically determining a person’s gaze point and tracking its movement over time. Over the years, eye trackers have evolved from invasive, costly, and technically demanding instruments into non-invasive tools that support various medical fields, including diagnostic interpretation, clinical assessment, and surgery [13]. By recording the gaze data of both eyes, eye-tracking technology enables the objective assessment and precise quantification of eye movement parameters, such as fixation stability, saccades, pursuits and vergence control [14].

Fixations are commonly analysed in terms of number, duration, and spatial distribution to provide insight into visual attention and information processing [9,10,15]. Saccadic movements can be evaluated using the variables such as frequency, amplitude (distance of movement), and duration, which help in understanding visual scanning efficiency [14]. Abnormal value of fixation and saccades may indicate underlying vision problems such as strabismus, which can be detected via eye tracker analysis through the demonstration of unstable fixation patterns [13]. As the clinical utility of these parameters has become more evident, researchers have explored the development of low-cost eye-tracking solutions and integrated software, making this technology more accessible for a broader range of applications and research.

This scoping review aims to explore and identify studies that investigate BVA in the paediatric population using different types of eye-tracking technology. Furthermore, it specifically reviews the application of eye tracking as a diagnostic tool for assessing visual disorders in children by analysing their eye movement patterns, such as fixation and saccades, which are commonly affected in conditions like strabismus and amblyopia [13].

## 2. Methodology

**Figure 1:**
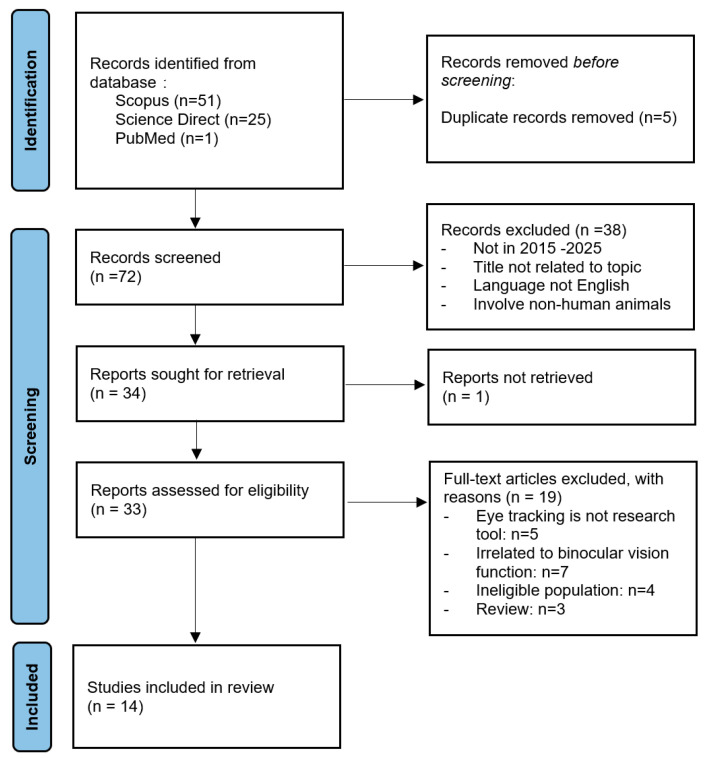
PRISMA-ScR flow diagram of study selection [16].

A comprehensive scoping review of the existing literature was conducted in accordance with the referred Reporting Items for Systematic reviews and Meta-Analyses extension for Scoping Reviews (PRISMA-ScR) reporting guidelines [16]. The PRISMA flow diagram (Figure 1) presents a summary of the study selection procedure.

**Eligibility Criteria:** Studies were selected based on predefined specific inclusion criteria. The target population comprised children and adolescence aged 0 to 18 years, including those with normal binocular functions as well as those diagnosed with BVAs [17]. Studies employed eye-tracking technology to assess binocular vision anomalies (e.g., strabismus, amblyopia, and phoria). The review focused on studies published between 2015 and 2025 to reflect recent progress in high-frequency binocular eye-tracking technology and analytical software. During this period, eye-tracking data analysis has been rapidly enhanced through automated gaze analysis, noise reduction, and pattern recognition [18,19]. These developments have enabled more precise and reliable identification of subtle gaze-vector differences which were challenging to detect in previous research. In addition, peer-reviewed journal articles published in English, including quantitative, qualitative, and mixed-method designs, were considered to ensure a comprehensive mapping of this specialised field. Conversely, studies were excluded if they involved adult populations (aged 19 years or older) or mixed-age groups without a discrete paediatric subgroup analysis. This exclusion criterion was implemented to ensure that the review remained strictly focused on the developmental nuances of paediatric visual coordination.

**Search Strategy:** The authors searched scientific databases such as Scopus, ScienceDirect, PubMed, and Google Scholar using a broad search approach that incorporated all fields and free text terms. The search terms included “eye-tracking,” “binocular,” “vision,” “anomalies,” “paediatrics,” and “children.” Table 1 presents the detailed search technique.

**Study Selection:** The inclusion and exclusion criteria were applied throughout the entire selection procedure. The review focused on the studies and reports published between 2015 and 2025. Titles and abstract were screened by the authors to ensure their relevance and adherence to the predefined criteria.

**Extraction and Charting of Results:** Data extraction forms were developed to systematically organise information related to the eye-tracking technologies used and the main metrics assessed. The extracted data were categorised into “Author(s) years,” “Type of Study,” “Subject,” “Device Used,” “Metrics,” “Assessment task,” and “Use of eye-tracking technology,” as presented in Appendix A. The PRISMA flowchart displayed in Figure 1 illustrates the database search process and the final selection of articles. The included studies were subsequently summarised based on their main characteristics.

## 3. Results

**Selection of Sources of Evidence:** The database search identified 51 articles from Scopus, 25 articles from ScienceDirect, and 1 article from PubMed, as summarised in Table 1. After the removal of duplicate articles, 72 articles remained for initial screening. These articles were screened according to their respective titles and abstracts, resulting in 33 articles being selected for full-text evaluation. Following the application of the inclusion and exclusion criteria, 14 articles were deemed eligible for the scoping review. Several studies were excluded as they did not focus on paediatric populations or did not assess binocular vision function. This study selection process is illustrated from the PRISMA flowchart presented in Figure 1.

**Characteristics of Source Evidence:** A total of 13 articles employed pupil-based eye tracking to evaluate eye movements, using devices such as the EyeLink 1000, Tobii, and ViewPoint ET. In addition, one study integrated eye-tracking system with Virtual Reality (VR) to measure the eye movements.

**Synthesised Results:** A total of 14 articles were categorised based on the type of eye-tracking technology used to assess eye alignment. The extracted information was presented according to the following categories: “Author(s) (years),” “Study Design,” “Subject,” “Device Used,” “Metrics,” “Assessment task,” and “Application of eye-tracking technologies,” as presented in Appendix A. Furthermore, the inclusion of these studies was supported by a more recent, updated literature that validated their methodologies and findings, ensuring that the review maintains a high degree of technical breadth and reliability.

According to the literature review, a variety of eye-tracking technologies have been used to analyse eye movement and alignment. These methodologies vary significantly in their approaches, ranging from wearable trackers to VR-based eye tracking. The integration of these technologies reflects a growing trend towards objective and automated measurement in clinical settings, allowing for more precise quantification of gaze deviations and stability metrics compared to traditional manual assessments methods.

## 4. Discussion

**Figure 2:**
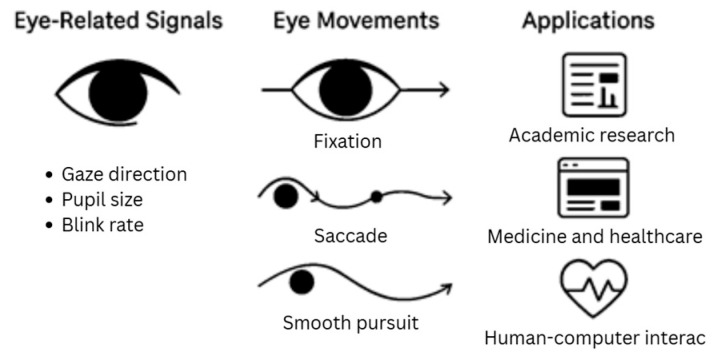
Signals, eye movements, and application of eye-tracking technologies [21].

This section focuses on the application of eye-tracking technology in diagnosing BVA. Despite growing interest in digital tools for vision evaluation, relatively few studies have addressed this topic over the past decade. The database search revealed a wide range of eye-tracking models and approaches used to assess eye movements and vergence. Eye-tracking systems typically capture multiple eye-related signals, including gaze direction, pupil size, and blink rate, while also enabling the analysis of key eye movements such as fixation, saccades, and smooth pursuit, as presented in Figure 2. These measurable parameters provide objective insights into oculomotor and binocular function, allowing the researcher to quantify the abnormalities that were not easily detected through clinical assessments. Beyond BVA research, eye-tracking technology has been applied more broadly to enhance vision assessment and expand the scope of vision care services, including early detection and potential prevention of ocular diseases. Furthermore, its applications have extended to academic research, medicine and healthcare, and human–computer interaction, highlighting its multidisciplinary relevance [5,13,20].

### 4.1. Application of Eye-Tracking in BVA

Modern eye-tracking technologies have been increasingly applied in both clinical and research settings to analyse the eye movements in children with BVAs. As outlined in Appendix A, the studies assessed fixation stability and saccadic behaviour to characterise eye-movement patterns and to compare their metrics between individuals with normal binocular vision and those with conditions such as amblyopia and strabismus. These findings suggested that eye-tracking can provide objective measurements of altered visual and oculomotor function associated with BVAs. However, relatively few studies have focused on paediatric populations diagnosed with conditions such as strabismus, amblyopia, and anisometropia [10,11,15,22,23,24,25,26]. These disorders often resulted in impaired visual coordination and suppression, both of which can be objectively assessed using eye-tracking tools. Notably, only one study investigated children with normal and abnormal phoria, highlighting a lack of proper methodological robust clinical research and indicating a gap in that could be addressed in future studies. Additionally, several studies included participants with nystagmus, a condition characterised by involuntary eye movements that can be precisely measured with an eye-tracking systems [23,27].

### 4.2. Remote/Screen-Based Trackers (Video-Based)

Eye-tracking technologies come in several types, such as screen-based remote trackers, head-mounted/wearable glasses, and VR integrated systems. Video-based infrared eye trackers, such as the EyeLink 1000 and EyeLink 1000 Plus, are widely regarded as the gold standard for clinical research due to their unparalleled precision and reliability. These devices consist of a fixed camera and a chin rest to stabilise the head position, enabling sampling rates of up to 2000 Hz. The high temporal and spatial resolutions make them particularly suitable for detecting subtle eye movement abnormalities, including fixation instability, vergence dysfunction, and saccadic disconjugacy [10]. Consequently, this has been extensively used in binocular vision research, particularly in studies investigating amblyopia and strabismus. The binocular vision eye movements were recorded, allowing for quantitative measurement of fixation instability, vergence abnormalities, and saccadic disconjugacy, providing detailed insights into binocular dysfunction in both anisometropic and strabismic amblyopia, as presented in Appendix A [10,11,22,23,28].

Several studies have demonstrated the clinical applicability of Eyelink systems in evaluating binocular vision disorders. Ghasia, Otero-Millan, and Shaikh (2018) utilised EyeLink 1000 to quantify fixation stability and saccadic disconjugacy in strabismus, revealing increased microsaccade amplitude, frequency, inter-saccade intervals, and inter-saccadic ocular drift [9]. The abnormalities are worse with larger deviations and absent stereopsis [9,29]. These parameters provide objective biomarkers of disrupted binocular fusion that complement cover testing and stereoacuity. More recent studies have integrated EyeLink systems into dichoptic viewing tasks, offering a refined method to assess binocular disparity and eye alignment under different visual input conditions [24,25]. Collectively, these studies demonstrate the robustness of EyeLink 1000 in advancing the understanding of binocular vision abnormalities. Despite their advantages, these high-precision systems require full cooperation from the patient, which can be challenging for young children to maintain head stability for extended periods. Additionally, high costs and the need for dedicated lab environments limit their widespread clinical adoption.

In addition, Tobii eye trackers have been increasingly used in clinical research due to their flexibility and non-invasive design. Tobii eye trackers are remote, screen-based devices that allow for greater freedom of head movement. This tolerance for natural head position makes them particularly suitable for studies involving paediatric populations. Several studies have demonstrated their application in binocular vision research. For example, the Tobii Eye Tracker 5 was used to examine fixational disparities in children with strabismic and normal binocular vision [30]. The Tobii EyeX has also been employed to record horizontal and vertical gaze points and convergence in individuals with normal and abnormal phoria, representing the only study specifically focused on phoria-related eye movement characteristics [31]. Likewise, the Tobii 1750 model was used to evaluate eye movement abnormalities in children with strabismus, both with and without amblyopia, across various experimental paradigms [26]. These applications demonstrate Tobii’s utility for screening and monitoring binocular disorders in routine clinical settings.

In summary, eye-tracking systems like EyeLink and Tobii exemplify complementary clinical tools for assessing binocular vison anomalies in amblyopia and strabismus. EyeLink offers superior precision through stationary, chin-rest setups with up to 2000 Hz sampling, ideal for detecting subtle fixation instability, saccadic disconjugacy, and vergence mechanisms in controlled research [9]. In contrast, Tobii’s remote, screen-based designs (60–120 Hz) tolerate natural head movements via head-box boundaries, enhancing paediatric suitability and ecological validity for screening fixation disparities, phoria, and natural-viewing abnormalities [26,30]. These differences in precision and flexibility influence how the systems are used clinically. EyeLink systems are typically used for detailed laboratory research, whereas Tobii systems are more practical for routine patient assessments.

### 4.3. Wearable/Head-Mounted Eye-Tracking Glasses

Wearable or head-mounted eye-tracking technology, such as the ViewPoint Eye Tracker, offers a lightweight and spectacle-style design. These head-mounted devices typically incorporate cameras positioned in front of each eye to track movements in real-world environments that are commonly used in sports, research, and navigation studies [32,33,34,35]. This approach supports binocular vision analysis by accompanying the participant, rather than requiring fixed positions. It is a unique approach on binocular vision by moving with the participant rather than requiring a fixed testing position. Cercenelli, Fresina, and Bortolani et al. (2018) demonstrated its effectiveness in evaluating fusional convergence responses, measuring peak velocity and saccadic behaviour under prism-induced stress or with real objects altering vergence demand [33]. The wearable eye tracker enables the assessment of binocular vision in more natural, ecological environments while improving participant comfort and testing conditions. However, this system may affect data analysis due to a lack of precision for interocular fixation instability, and it fits poorly over prisms or thick lenses worn by patients with binocular vision disorders.

The EyeSeeCam video-oculography system (Munich, Germany) has been utilised in vestibular Head Impulse Testing (vHIT) [34]. This device allows for the changing of the camera’s location during both monocular and binocular recordings. The vHIT is reliable for evaluating the high-frequency VOR of the six semicircular canals and is generally more comfortable than older vestibular tests, such as the bedside HIT (Halmagyi–Curthoys test) [35]. The raw vHIT data were reviewed for the presence of covert or overt saccades. Despite this, the study found that the system presented no consistent detection of overt or covert saccades in patients with acquired esotropia [34]. This funding suggests that although saccades are generally part of oculomotor assessment, they were not a primary indicator of esotropia in this specific study.

Overall, wearable eye-tracking systems offer a naturalistic approach to evaluating binocular vison and oculomotor response in dynamic environments, providing insights into vergence and coordination during real-world tasks. The limitations in precision, compatibility with corrective lenses/prisms, and data-processing complexity may affect sensitivity for subtle anomalies in clinical applications. A comprehensive summary of studies utilising the ViewPoint Eye Tracker and the EyeSeeCam video-oculography system is provided in Appendix A.

### 4.4. VR-Based Eye Tracking

VR devices incorporating eye-tracking technology have been developed and applied in clinical practice. Cameras embedded within VR headsets to record eye movements during both interaction and analysis in virtual environments, enabling simultaneous assessment of visual behaviour and user responses within controlled stimuli. Recent studies demonstrate that strabismus measurements from VR devices correlate moderately with gold-standard sensorimotor examinations, with stronger correlations for esotropia and constant deviations [36].

By integrating infrared-based eye-tracking software into head-mounted displays, these systems allow for the assessment of eye position and the detection of vision disorders such as strabismus. Notable examples include the Tobii-integrated VR headsets and Olleyes VisuALL ETS VR headsets which using Olleyes software [36]. Mori et al. reported that earlier VR systems relied on infrared sensor-based eye tracking, whereas more recent approaches combine VR with artificial intelligence (AI), achieving up to 83% sensitivity through real-time pupil and iris recognition and providing detailed raw eye movement data (Appendix A) [37]. The AI-VR system demonstrated high sensitivity for detecting strabismus and ocular deviations and strong correlations to gold standards (R = 0.76 near esotropia mode). In comparison, inter-method agreement varied by deviation type, ranging from high for near esotropia (ICC = 0.82) to low for exotropia (ICC < 0.4) [37].

In summary, these advances demonstrate the progression of eye-tracking technology from sensor-based detection to AI-driven analysis, offering valuable insights into binocular coordination during controlled tasks. Nonetheless, further development is needed before these systems can be fully implemented in clinical settings. One remaining challenge is the detection of exotropia, as precise measurement of the angle in VR is still hindered by factors such as “proximal convergence,” whereby the eyes natural tendency to turn inward (converge) is due to the perceived or physical closeness of the VR display to the face [7].

### 4.5. Devices Used and Their Characteristics

According to the studies reviewed, the researchers employed a range of eye-tracking systems, which include remote/screen-based trackers, wearable/head-mounted eye-tracking glasses, and VR-based eye tracking. A detailed comparison of these modalities, including their technical specifications and clinical utility, has been provided in Appendix B.

The screen-based eye trackers (like EyeLink 1000 and EyeLink 1000 Plus) have high precision in tracking gaze on two-dimensional targets due to their high sampling rates (up to 2000 Hz), sub-minute accuracy, and low latency. These technologies have been prominently featured due to their advanced capabilities in detecting subtle oculomotor issues in BVA, including fixation instability, saccadic disconjugacy, and vergence anomalies. Still, their setup demands a dedicated computer, specialised mount, and chin rest, which limits portability and convenience outside controlled environments. Consequently, screen-based systems are ideally suited for controlled clinic or laboratory settings rather than real-world or field-based applications.

In addition, wearable eye trackers (e.g., EyeX, 1750, and Eye Tracker 5) allow for the assessment of natural visual behaviours, making them well-suited for dynamic tasks such as reading and object tracking and particularly appropriate for paediatric studies. For instance, ViewPoint Eye Tracker can provide valuable insights into fusional convergence and intrusive saccades under binocular stress conditions. These devices typically require only specialised goggles or a tight-fitting headband to capture gaze data during real-world tasks. This is advantageous in diagnosing and monitoring vestibular disorders, as well as behavioural and clinical eye-movement studies. However, they generally offer lower precision compared to screen-based systems, with trade-offs in spatial accuracy and sampling rates.

VR systems, such as Olleyes VisuALL ETS, support both diagnosis and treatment through dichoptic stimulation and precise eye alignment measurements. These systems have demonstrated moderate-to-strong correlation with traditional clinical tests, particularly in assessing constant esotropia [36]. Although they only have moderate accuracy compared to screen-based eye tracking, VR systems deliver immersive 3D environments and perfect control over visual stimuli, which are crucial for dynamic assessments. They are commonly applied in neuro-rehabilitation, phobia therapy, and visual field testing. Conversely, potential drawbacks include motion sickness and the bulky nature of the headsets.

In summary, technological innovations have significantly enhanced the precision and flexibility of oculomotor assessment. Screen-based (remote) systems offer high spatial accuracy for detailed fixation and saccade analysis whereas wearable devices offer superior ecological validity for capturing real-world visual behaviours. VR-based systems further complement these approaches by providing controlled and immersive environments for assessing binocular function. This review demonstrates that screen-based and VR eye trackers are particularly suitable for precise clinical assessments, while wearable trackers are recommended for efficient, large-scale vision screening in community settings, enabling natural eye movement capture during everyday tasks. Therefore, these technologies serve as the foundation for binocular vision function assessment, with the optimal choice depending on the setting (e.g., clinical or field) and population involved.

## 5. Limitations

There are several notable limitations to this scoping review. The inclusion of only 14 relevant studies indicates a relatively limited evidence base, which may restrict the generalisability of the conclusions. There is also methodological variability in terms of data quality, resolution, and outcome measures due to the use of different eye-tracking systems across the literature. Furthermore, this review may be subject to language and publication bias as only English-language and peer-reviewed sources were included. In addition, potentially valuable insights may have been omitted due to the exclusion of grey literature, such as conference proceedings and unpublished data.

These review-specific limitations reflect broader trends in the field, where comprehensive clinical research remains sparse despite promising technological advancements. In particular, non-strabismic binocular vision abnormalities like convergence insufficiency are significantly underexplored in eye tracking research. These gaps indicate the importance of further clinical studies, as well as the development of standardised procedures and validated instruments to ensure the consistent and reliable implementation of eye-tracking technologies in clinical practice.

## 6. Conclusions

This scoping review highlights the development and clinical potential of eye-tracking technology as a promising approach for assessing BVA, particularly in paediatric populations. Various eye-tracking systems, including remote/screen-based eye tracking, wearable eye tracking, and VR-based eye-tracking systems, have been employed to analyse eye movement patterns associated with these anomalies. These advanced technologies provide objective and precise measurements of oculomotor function, thereby enhancing both diagnostic accuracy and monitoring of therapeutic interventions.

Beyond improved diagnostics, this review underscores eye-tracking’s role in uncovering the underlying mechanisms causing BVA in children, paving the way for focused interventions that address root causes rather than symptoms alone. By bridging gaps in paediatric eye care, these innovations have the potential to raise public awareness of childhood eye health. This makes advanced examinations more accessible and expands preventive treatments to underserved communities. Ultimately, this study demonstrates the transformative significance of eye-tracking in the early detection and management of BVA, with profound implications for lifelong visual health, educational outcomes, and reduced healthcare burdens.

In conclusion, further research is necessary to broaden the scope of existing eye-tracking technologies, increase their reliability, and establish their role in standard clinical practice. Nevertheless, eye tracking holds considerable promise for advancing the assessment and comprehension of binocular vision abnormalities. In response, future investigations incorporating diverse populations, standardised methodologies, and real-world clinical settings will be crucial in fully realising the potential of eye-tracking technologies in vision science and clinical care.

## Figures and Tables

**Table 1:** Search strategy on eye-tracking technologies in binocular vision anomalies.

No.	Databases	Applied Keywords and Boolean Operators
1	Scopus—51	“eye-tracking” AND “binocular” AND “vision” AND “anomalies” AND “paediatrics” AND “children.”
2	Science Direct—25	
3	—PubMed—1	

## Data Availability

The original contributions presented in this study are included in the article. Further inquiries can be directed to the corresponding author(s).

## Appendix A. Summary of Eye-Tracking Technologies in Assessing Binocular Vision Anomalies

**Author(s) (Year)**	**Study Design**	**Subjects**	**Device Used**	**Metrics**	**Assessment Task**	**Application of** **Eye-Tracking Technologies**
Ghasia & Tychsen (2024) [23]	Experimental study	47 controls (15.3 ± 12.2 years of age) and 104 amblyopic subjects (13.3 ± 11.2 years of age)	EyeLink 1000 Plus	- Fixation stability - Vergence stability - Inter-ocular stability	- A white circular target (0.5° visual angle) projected against a black background	- Eye tracking measures the horizontal and vertical eye positions - Amblyopic individuals exhibit increased fixation instability compared to controls - Nystagmus was present in 27% of anisometropic amblyopes and over 65% of strabismic/mixed amblyopes; younger age and presence of nystagmus further increased fixation and vergence instability (*p* < 0.05)
Quagraine et al. (2024) [25]	Experimental study	27 amblyopic and 8 healthy control participants	EyeLink 1000 Plus	- Fixation duration - Fixation stability	- A dot with (0.5 degrees visual angle)	- Eye tracking measured deviations in eye positions during dichoptic viewing tasks - Provide insights into visual attention dynamics of fellow eye and amblyopic eye
Upadhyaya et al. (2025) [28]	Observational study	Amblyopia group: a mean age of 13 years Control group: mean age of 14.90 years	EyeLink 1000 Plus	- Fixation stability - Microsaccades - Drifts	- A white circular target (with black background)	- Fixation eye movements are recorded when subject focused at the stimulus - Cartoon images and audio sounds - The binocular viewing, fellow eye viewing, and amblyopic eye viewing were tested to determine how these conditions influenced fixation instability
Murray et al. (2022) [24]	Prospective, controlled laboratory study	34 patients with amblyopia & 7 healthy controls	EyeLink 1000	- Fixation stability - Fixational saccades - Inter saccadic drifts	- A white circular target with 0.5 deg visual angle	- Subjects were required to fixate a circular target under different monocular, binocular, and dichoptic viewing conditions - Fixation eye movements in amblyopia patients show different outcomes depending on the viewing conditions - Patients with strabismic/mixed amblyopia without fusion maldevelopment nystagmus has a higher likelihood to exhibit a fixation switch
Kelly et al. (2019) [10]	A prospective, comparative study	160 children age 4–12 years with treated esotropia and/or anisometropic	EyeLink 1000	- Fixation stability - Saccades - Vergence stability	- A white dot with 0.3 deg diameter	- Fixation instability was recorded and quantified using the bivariate contour ellipse area - From the eye tracking data, it has showed an increase in fixation and vergence instability in children with strabismus and anisometropia, as well as children with amblyopia
Chen et al. (2018) [11]	Experimental study	31 children (controls = 10; amblyopia = 21)	EyeLink 1000	- Microsaccades - Saccades - Intersaccadic drifts - Slow phase velocities (in patients with latent nystagmus)	- A red circular target (with 0.5 visual angle)	- Reduce frequencies of microsaccades and saccadic movement in amblyopia and subjects who may develop of latent nystagmus
Ghasia, Otero-Millan & Shaikh (2018) [9]	Cross-sectional study	13 patients with strabismus and 16 controls	EyeLink 1000	- Fixation stability - Intersaccadic drift velocity - Fixational saccades - Amplitude of saccades - velocity of saccades	- Visual fixation: a red circular visual target - Visual search: to identify differences (10 total) between two similar looking images and click on them.	- Fixation instability was greater in the deviating eye for patients with strabismus - Strabismic patients show a higher intersaccadic drift velocity in both viewing and non-viewing eye - Strabismic patients has a greater disconjugacy in the fixational saccades, visually guided saccades and intrasaccadic eye drifts
Shaikh, Otero-Millan Kumar & Ghasia (2016) [15]	Cross-sectional study	11 healthy children and 36 paediatric subjects with amblyopia	EyeLink 1000	- Fixational - Microsaccades - Saccadic drift	- A circular target with 0.5 visual angle	- Eye tracker measures the horizontal and vertical eye positions while the subject focused the target for 45 s - Provide insights into the mechanisms underlying fixation instability in amblyopia and suggest that the abnormalities in fixational saccades are likely due to disruptions in the neural architecture of the visual system.
Hou, Yang, Chen & Liao (2025) [30]	A quantitative, observational study	4–18 years old participant with strabismic and controls	Tobii eye tracker 5	- Fixation disparity	- Dichoptic Fixational Disparity Test - Bright blue dots (1.4 deg diameter)	- Used to record binocular gaze positions using polarised glasses - Strabismic children exhibited larger horizontal and vertical fixational disparities compared to normal children - Fixational function is poorer in children with esotropia compared to those with exotropia
Kim et al. (2022) [31]	Experimental study	35 abnormal phoria & 25 normal phoria	Tobii EyeX	- Fixation stability - Convergence	- A red dot target 3.7 mm (with black background)	- Subjects were instructed to fixate on the target for 15 s (to assess fixation stability) - Using an eye tracker to distinguish the difference in abnormal and normal phoria for non-strabismus patients - Fixation stability was found to be associated with convergence
Mutlu et al. (2022) [34]	A prospective case–control study	17 accommodative esotropia, 24 non-accommodative esotropia, & 21 healthy controls	video head impulse test (vHIT) & functional head impulse test (fHIT)	- VOR gain - saccades	- A coloured sticker	- Using vHIT but cannot used to differentiate the VOR gain and saccades between normal and abnormal group - The raw vHIT data was reviewed to check for the presence of covert or overt saccades - Using fHIT with large score found in healthy group
Al-Haddad et al. (2019) [26]	Prospective observational case–control study	Children aged 7–17 years: 50 with strabismus (24 with amblyopia); 50 controls	Tobii 1750	- Latency (time to first fixation) - First fixation duration - Total fixation duration - Fixation count - Percentage of fixation	- Distance and near paradigms - Reading task - Location Identification Paradigm - Moving Target Paradigm	- Providing a comprehensive analysis of various eye movement parameters, including latency to first fixation, first fixation duration, and total fixation count - Children with strabismus exhibit significant eye movement abnormalities compared to controls, especially in tasks involving reading and identifying key elements - Abnormalities are more marked in children with strabismus without amblyopia
Cercenelli et al. (2018) [33]	Quantitative approach	26 subjects with normal binocular vision	ViewPoint ET	- Fusional convergence - Convergence velocity - Intrusive saccades - Saccadic amplitude	- A 0.5° black fixation dot on a white background - Based-out prism	- Eye tracking with Saclab toolbox to analyse convergence duration, peak convergence velocity and the amplitude of intrusive saccades - Increased of convergence velocity can be due to high disparities - Intrusive saccadic and mean saccadic amplitude increase as higher prism magnitude (higher disparities)
Mori et al. (2025) [36]	Prospective, masked diagnostic test study	Patients aged 5–18 years	VR-based eye-tracking technology	- Eye deviation angle	- The red stimulus is alternatively presented monocularly	- To measure eye position and alignment - VR-based simulated alternate cover test - Strongest correlation between patients with esotropia or constant deviations - Strabismus measurements collected using the VR device showed significant correlation with results from the standard sensorimotor examination

## Appendix B. Different Types of Eye-Tracking Technologies and Their Performance Characteristics

**Category**	**Specific Devices**	**Type of Technology**	**Accuracy (Visual Angle)**	**Setup Complexity**	**Calibration Demands**	**Primary Advantages**	**Significant Limitations**	**Ideal Use Case**
Remote/Screen-Based Eye Trackers	Infrared Video-Based (EyeLink 1000, 1000 Plus) *	Fixed-Mount PCCR * (High-Speed CMOS)	Highest (<0.15–0.5°)	High: Requires host PC, specific mounts, and chin rest	High: Multi-point (9–13 pts); highly sensitive to head drift.	Gold standard for precision; extremely low latency	Expensive; bulky; requires fixed head position (for max accuracy)	Neuro-scientific research, reading studies, and micro-saccades
	Remote Video-Based (Tobii EyeX, Tobii 1750, Tobii 5) *	Remote PCCR (Monitor-mounted)	Moderate (~0.5–1.0°)	Low: Mostly “plug-and-play” USB bars or integrated screens	Low-Moderate: Rapid 1–9 point calibration	Unobtrusive; allows for natural head movement; easy for children/elderly.	Lower sampling rates (30–120 Hz)	Clinical screening, UX testing, and developmental psychology
Head-Mounted/Wearable Eye Trackers	vHIT (video Head Impulse Test) *	High-Speed VOG (Goggles)	Clinical (VOR Gain Error < 5%)	Moderate: Requires specialised goggles and tight fitting	High: Requires “geometric” & “gain” calibration for reflex accuracy	Objective assessment of vestibulo-ocular reflex	Limited to vestibular	Diagnosis and monitoring of vestibular disorders
	ViewPoint Eye tracker	Head-mounted VOG	Moderate (~0.5–1° depending on configuration)	Moderate	Moderate calibration	Allows for free head movement; suitable for natural viewing tasks	Lower spatial precision than fixed systems	Behavioural and clinical eye-movement studies
VR / AR-Integrated Eye Trackers	VR-Based ET (Tobii VR, Olleyes VisuALL ETS) *	HMD-Integrated VOG	Moderate (~0.5–1.1°)	Moderate: Integrated into a headset; requires VR PC/Stand-alone	Low: Headset-based calibration	Immersive 3D environments; perfect control over visual stimuli.	Potential for motion sickness; bulk of the headset	Neuro-rehabilitation, phobia therapy, and visual field testing
* Eye-tracking technologies included in the 14 articles of scoping review.								
